# Rigidez Arterial e Funções Miocárdicas do Ventrículo Esquerdo em Crianças com Válvula Aórtica Bicúspide Funcional

**DOI:** 10.36660/abc.20200657

**Published:** 2021-09-03

**Authors:** Pelin Kosger, Tugcem Akin, Hikmet Kiztanir, Birsen Ucar

**Affiliations:** 1 Eskisehir Osmangazi University Faculty of Medicine Department of Pediatric Cardiology Eskisehir Turquia Eskisehir Osmangazi University, Faculty of Medicine, Department of Pediatric Cardiology, Eskisehir - Turquia; 2 Eskisehir State Hospital Pediatric Cardiology Clinic Eskisehir Turquia Eskisehir State Hospital, Pediatric Cardiology Clinic, Eskisehir - Turquia

**Keywords:** Aorta, Rigidez Aórtica, Dilatação Patológica, Função Ventricular Esquerda, Análise da Onda de Pulso, Miocárdio; Criança

## Abstract

**Fundamento:**

A rigidez arterial é um importante preditor de aortopatia e remodelamento miocárdico em pacientes com válvula aórtica bicúspide, podendo estar aumentada na infância.

**Objetivo:**

Avaliar a rigidez arterial e a função miocárdica do ventrículo esquerdo em crianças com válvula aórtica bicúspide funcional.

**Métodos:**

Quarenta e quatro crianças com válvula aórtica bicúspide e 41 pares saudáveis com válvula aórtica tricúspide foram incluídos neste estudo caso-controle. Foram obtidos os diâmetros e os escores-z relacionados da raiz aórtica e da aorta ascendente. Quanto à função miocárdica do ventrículo esquerdo, juntamente com as velocidades de fluxo mitral e parâmetros do Modo M, as velocidades miocárdicas e os intervalos de tempo foram avaliados com Doppler tecidual. A análise da onda de pulso foi realizada por aparelho oscilométrico (Mobil-o-Graph). Um valor de p<0,05 foi considerado significativo.

**Resultados:**

O índice da massa ventricular esquerda, a velocidade A do fluxo mitral, o diâmetro e o escore z da aorta ascendente e o índice de desempenho miocárdico estavam significativamente maiores nos pacientes (p = 0,04, p = 0,02, p = 0,04, p <0,001 e p <0,001 respectivamente). O índice de desempenho miocárdico correlacionou-se positivamente com o diâmetro da aorta ascendente e a velocidade A (r=0,272; p=0,01, r=356; p=0,001, respectivamente). A análise multivariada revelou que o índice de desempenho miocárdico estava relacionado ao diâmetro da aorta ascendente (p = 0,01). O índice de aumento e a velocidade da onda de pulso foram semelhantes entre os grupos (p> 0,05).

**Conclusão:**

De acordo com a análise da onda de pulso oscilométrico, as crianças com válvula aórtica bicúspide funcional apresentam rigidez arterial semelhante a seus pares saudáveis. O diâmetro da aorta ascendente foi estabelecido como preditor independente da função miocárdica do ventrículo esquerdo. A rigidez arterial pode não ser um fator de risco grave em pacientes pediátricos sem dilatação acentuada da aorta ascendente.

## Introdução

A válvula aórtica bicúspide é a malformação cardíaca congênita mais comum e ocorre em 1-2% da população geral. ^1^ Além da função valvar diminuída, os indivíduos também estão sob risco de aortopatia, que pode resultar em dissecção aórtica e formação de aneurisma. ^2^^,^^3^ Não há associação direta entre disfunção valvar e aortopatia. ^4^ A presença de dilatação da aorta, mesmo em pacientes com função valvar normal, pode estar relacionada a uma fisiopatologia diferente da aortopatia. ^5^ Em comparação com controles saudáveis, pacientes com válvula aórtica bicúspide sem disfunção valvar aparente têm mostrado elasticidade aórtica diminuída e rigidez aórtica central aumentada. ^6^ Além disso, nesses indivíduos, a rigidez aórtica central, medida com o método de monitorização ambulatorial da velocidade da onda de pulso, está positivamente correlacionada com o grau de dilatação aórtica. ^7^ Portanto, o remodelamento vascular tem sido identificada como a principal causa de rigidez arterial que afeta o processo de monitorização em pacientes. ^8^ O nível de rigidez arterial, um preditor do curso de doenças cardiovasculares, tende a aumentar com a idade. Entretanto, o exame de amostras de biópsia intraoperatória e necropsia mostraram que em muitas doenças cardíacas congênitas, a rigidez arterial aumenta desde a infância. ^9^^,^^10^ Pacientes com válvula aórtica bicúspide têm dilatação aórtica progressiva durante a infância, e os dados sobre elasticidade aórtica e rigidez arterial são baseados em métodos ultrassonográficos, com número bastante limitados. ^11^^,^^12^ O método padrão ouro para medir a rigidez arterial é a análise da onda de pulso, e a técnica de análise de onda de pulso mais comumente utilizada é a tonometria; entretanto, essa técnica pode ser demorada e desafiadora, especialmente quando utilizada em crianças mais jovens. ^13^ Os dispositivos oscilométricos são fáceis de usar, práticos para uso em ambiente clínico e um método confiável para avaliar a pressão arterial central e os parâmetros de rigidez arterial, mesmo em crianças. ^14^^,^^15^

Os pacientes com válvula aórtica bicúspide também sofrem de remodelamento miocárdico, independentemente das funções valvares e da aortopatia. Entretanto, para confirmar que o remodelamento miocárdico não está associado apenas às funções valvares, são necessários estudos que incluam casos com funções valvares intactas sem fatores de risco adicionais. Os casos pediátricos constituem um grupo ideal de pacientes para esse fim, e atualmente existem poucos estudos na literatura relatando funções diastólicas do ventrículo esquerdo afetadas em pacientes pediátricos. ^12^^,^^16^ Nosso objetivo foi avaliar a rigidez arterial utilizando o método oscilométrico e determinar sua compatibilidade com o nível de elasticidade arterial ultrassonográfica em crianças com válvula aórtica bicúspide funcional. Além disso, objetivamos avaliar a função miocárdica global através do índice de desempenho miocárdico, derivado da imagem de Doppler tecidual em nosso estudo.

## Materiais e Métodos

### População de estudo

Um total de 44 pacientes (7 a 18 anos de idade) com diagnóstico de válvula aórtica bicúspide, acompanhados na clínica de cardiologia pediátrica foram incluídos no estudo. Esses pacientes não apresentavam disfunção valvar aórtica aparente, nem recebiam medicação preventiva para aortopatia. Quarenta e uma crianças com válvulas aórticas tricúspides saudáveis e com características demográficas e antropométricas semelhantes foram incluídas como grupo controle. Foram excluídos do estudo pacientes com insuficiência valvar moderada a grave, apresentando velocidade valvar > 2m/s, pacientes submetidos a cirurgia anterior ou intervenção percutânea e com doença cardíaca adicional submetida a reparo ou não (por exemplo, coarctação da aorta), bem como aqueles com índice de massa corporal e pressão arterial sistólica > percentil 95. Além disso, pacientes que receberam medicação preventiva não foram incluídos para evitar os efeitos confundidores dos medicamentos sobre a mecânica miocárdica e a rigidez arterial. Conforme demonstrado por testes laboratoriais, nenhuma das crianças da população do estudo apresentavam hipercolesterolemia. Os pacientes deram consentimento informado por escrito para o estudo e o estudo foi aprovado pelo Comitê de Ética local da Eskisehir Osmangazi University em 04 de abril de 2018.

### Ecocardiografia

A ecocardiografia transtorácica foi realizada por um único cardiologista pediátrico experiente utilizando o equipamento disponível comercialmente Affinity 70 (Philips Medical Systems, Bothell, WA, EUA) com sondas de banda larga de 2–4 e 4–8 MHz. O exame vascular foi realizado através de ultrassonografia com Doppler colorido utilizando o equipamento VIVID I (General Electric Ultrasound Systems, Mountain View, CA, EUA), com uma sonda linear de 12 MHz.

#### Avaliação da morfologia e funções da válvula aórtica

A morfologia da válvula aórtica foi avaliada nos cortes paraesternais de eixo longo e curto. A frequência da sonda foi selecionada de acordo com o tamanho do paciente. A válvula aórtica bicúspide definitiva foi diagnosticada quando apenas dois folhetos valvares foram inequivocamente identificados na sístole e na diástole, com uma aparência nítida de “boca de peixe” na sístole. O fenótipo do folheto foi definido como anteroposterior (fusão das cúspides esquerda e direita) ou direita-esquerda (fusão das cúspides direita e não coronária). Para avaliação da estenose da válvula aórtica, o pico de velocidade aórtica foi medido utilizando Doppler de ondas contínuas enquanto o cursor era mantido no nível do seio de Valsalva no corte de cinco câmaras, sendo um valor > 2,5 m/s considerado indicativo de estenose aórtica. ^17^ Para determinar o grau de insuficiência valvar aórtica, foi utilizada a proporção do diâmetro da insuficiência aórtica medido nas imagens do Doppler colorido, obtidas no corte de eixo longo, em relação ao diâmetro da via de saída do ventrículo esquerdo. Proporções <25%, de 25 a 64% e ≥65% geralmente indicam regurgitação aórtica leve, moderada e grave, respectivamente. ^18^

#### Medida da raiz aórtica e diâmetro da aorta ascendente

As medidas dos quatro segmentos aórticos, incluindo o anel aórtico, seio de Valsalva, junção sinotubular e aorta ascendente proximal 2 cm acima da junção sinotubular, foram obtidas no corte paraesternal de eixo longo no final da diástole, na interface das bordas e perpendicular ao eixo longo da aorta. ^19^ As dimensões da aorta foram normalizadas para a área de superfície corporal. Um escore-z >2 foi considerado anormal.

#### Ecocardiografia com Doppler tecidual e no Modo M do ventrículo esquerdo

As dimensões internas do ventrículo esquerdo, a espessura do septo interventricular e a espessura da parede posterior foram medidas no final da diástole utilizando ecocardiografia bidimensional no modo M de acordo com as diretrizes pediátricas da *American Society of Echocardiography* . ^20^ A massa ventricular esquerda foi calculada de acordo com a Fórmula de Devereux ^21^ e indexada à estatura. A fração de ejeção do ventrículo esquerdo foi calculada pela fórmula de Teichholz. ^22^ O volume da amostra do Doppler foi posicionado nas pontas dos folhetos mitrais para obter os formatos de onda de fluxo do ventrículo esquerdo no corte apical de quatro câmaras. A velocidade diastólica inicial do fluxo mitral (E) e a velocidade diastólica tardia (A) também foram medidas. Para medir as velocidades miocárdicas longitudinais, o volume da amostra foi colocado no segmento septal do anel mitral para obter os formatos de onda do corte apical de quatro câmaras. A velocidade diastólica inicial do anel mitral (Ea) e a velocidade diastólica tardia do anel mitral (Aa) foram medidas e sua razão (Ea/Aa) foi calculada para estimar a pressão de enchimento ventricular esquerdo. Os intervalos de tempo cardíaco, incluindo o tempo de relaxamento isovolumétrico, tempo de contração isovolumétrica e tempo de ejeção, foram obtidos por imagens de Doppler tecidual e o índice de desempenho miocárdico foi calculado de acordo com a fórmula de Tei: índice de desempenho miocárdico = (tempo de contração isovolumétrica + tempo de relaxamento isovolumétrico)/tempo de ejeção. ^23^ Para cada parâmetro quantitativo, foi calculada a média de três batimentos consecutivos.

#### Avaliação da elasticidade aórtica

A elasticidade aórtica foi avaliada utilizando o modo M guiado pelo ecocardiograma bidimensional dos diâmetros aórticos sistólico e diastólico 2 cm acima da válvula aórtica; o diâmetro diastólico foi obtido no pico da onda R no eletrocardiograma registrado simultaneamente, enquanto o diâmetro sistólico foi medido no movimento anterior máximo da parede aórtica.

A deformação miocárdica (strain), a distensibilidade aórtica e o índice de rigidez aórtica foram calculados utilizando as fórmulas abaixo:

*Deformação miocárdica* = 100 (Diâmetro sistólico-Diâmetro diastólico)/Diâmetro diastólico

*Índice de rigidez aórtica* = Logaritmo natural (Pressão arterial sistólica/ Pressão arterial diastólica) / [(Diâmetro sistólico-Diâmetro diastólico/Diâmetro diastólico]

*Distensibilidade aórtica* (cm^2^/dyn/10^-6^) = 2x (Diâmetro sistólico-Diâmetro diastólico) / [(Pressão arterial sistólica – Pressão arterial diastólica) X Diâmetro diastólico] ^24^

### Medida da rigidez arterial

Para análise da onda de pulso e monitorização da pressão arterial, foram utilizados o dispositivo Mobil-O-Graph (IEM, Industrielle Entwicklung Medizintechnik und Vertriebsgesellschaft mbH, Stolberg, Alemanha) e o *software* de análise de onda de pulso ARCSolver (AIT Austrian Institute of Technology GmbH, Viena, Áustria). A monitorização da pressão arterial de 24 horas foi realizada conectando-se um manguito de tamanho apropriado para a circunferência do braço. Durante o teste, foram medidos a pressão arterial sistólica periférica e central, pressão arterial diastólica periférica e central, pulso, velocidade da onda de pulso e índice de aumento.

O Mobil-O-Graph é um aparelho oscilométrico utilizado na medida ambulatorial da pressão arterial, apropriado para uso em crianças. ^25^^,^^26^ Após medir a pressão arterial, o manguito é inflado até o nível da pressão diastólica braquial e as oscilações (ondas de pulso) são registradas por 10 segundos. Após o tempo de medição de 24 horas, todas as medidas são transferidas para o *software* HMS Client e analisadas com o *software* ARCSolver, já aplicado em crianças. ^27^

A velocidade da onda de pulso aórtica e o índice de aumento na frequência cardíaca (FC) de 75 batimentos/minuto (AIx @ 75) são marcadores de rigidez arterial. ^28^ A velocidade da onda de pulso aórtica é a velocidade na qual as ondas de pulso viajam na parede aórtica e constitui uma medida da rigidez arterial central. O AIx @ 75 é derivado da pressão de aumento e da pressão de pulso de uma onda de pulso. A onda de pulso é uma soma das ondas diretas (produzindo o primeiro pico sistólico) e refletidas (produzindo o segundo pico). O aumento na amplitude da onda de pulso devido ao reflexo da onda de pulso é conhecido como aumento de pulso e sua contribuição para a amplitude da onda de pulso é conhecida como pressão de aumento. Além disso, a porcentagem da amplitude da onda de pulso devido à pressão de aumento é conhecida como índice de aumento, que depende da frequência cardíaca. O Mobil-o-Graph fornece o índice na frequência cardíaca de 75bpm, uma medida de rigidez arterial periférica. ^29^

### Análise Estatística

A análise estatística foi realizada utilizando-se o programa *Statistical Package for Social Sciences* 18 (SPSS, Chicago, EUA). O tamanho da amostra foi determinado pelo *software* de análise G-power, com um poder estatístico de 85%. O teste de Kolmogorov-Smirnov foi utilizado para avaliar a distribuição normal. Os resultados das variáveis contínuas foram expressos em média ± desvio padrão (DP) ou mediana (percentil 25 e 75, intervalo interquartil, IIQ). Os grupos foram comparados com o teste T de amostras independentes para variáveis contínuas, e o teste U de Mann-Whitney foi utilizado para variáveis com distribuição não normal. O teste do qui-quadrado foi utilizado para a comparação de gênero entre os grupos. O teste de correlação de Spearman foi utilizado para correlações. Foi realizada a análise de regressão linear múltipla pelo método *Backward* para avaliar o preditor independente do índice de desempenho miocárdico em pacientes com válvula aórtica bicúspide. O nível de significância estatística foi estabelecido com p <0,05.

## Resultados

A mediana de idade de toda a população do estudo (n = 85) foi de 12 (IQR = 8,5–14) e variou de 7 a 18 anos. Doze pacientes no grupo com válvula aórtica bicúspide (12/44) e 18 casos do grupo controle (18/41) eram do sexo feminino (p = 0,084). Não houve diferenças significativas em relação a idade, peso, altura, área de superfície corporal, índice de massa corporal, perfil lipídico sérico e níveis de glicose entre os grupos ( Tabela 1 ).

**Tabela 1:** Características basais demográficas, antropométricas e clínicas dos grupos

Variável	Pacientes (n = 44)	Controles (n = 41)	p-valor
Idade (anos)	12 (9 - 15)	12 (8 -14)	0,609
Sexo (feminino,%)	12 (27,27)	18 (43,90)	0,084
Altura (cm)	147,8 ± 20,1	146,8 ± 15,5	0,798
Peso (kg)	42,3 ± 16,4	38,6 ± 14	0,268
IMC (kg/m^2^)	18 ± 0,3	17 ± 0,3	0,088
CT (mg/dl)	141,1 ± 23,1	148,3 ± 28,4	0,308
TG (mg/dl)	91,6 ± 38,9	86,6 ± 31,9	0,113

*IMC: índice de massa corporal; CT: colesterol total; TG, triglicérides totais. Variáveis contínuas com distribuição normal são expressas como média ± desvio padrão, e aquelas com distribuição não normal como mediana (intervalo interquartil)* .

Os parâmetros ecocardiográficos e de elasticidade aórtica estão resumidos nas Tabelas 2 e 3 . A velocidade A do fluxo mitral, o tempo de relaxamento isovolumétrico, o tempo de contração isovolumétrica e o índice de desempenho miocárdico foram significativamente maiores nos pacientes (p = 0,03, <0,001, <0,001, <0,001, respectivamente). A fusão das cúspides coronárias esquerda e direita, definida como fenótipo anteroposterior, foi o fenótipo predominante (63,6%). Junto com a velocidade aórtica, o diâmetro da aorta ascendente e o escore-z foram maiores nos pacientes (p <0,001, p = 0,04, p <0,001, respectivamente). Todos os parâmetros de elasticidade aórtica foram semelhantes entre os grupos. As variáveis hemodinâmicas centrais e periféricas, Alx@75 e valores de velocidade da onda de pulso, que não mostraram diferença significativa entre os grupos, são apresentadas na Tabela 4

**Tabela 2:** Medidas ecocardiográficas em pacientes e controles

Variável	Pacientes (n = 44)	Controles (n = 41)	p-valor
DSIV (mm)	6,9 ± 1,0	6,7 ± 0,9	0,429
DDFVE (mm)	45 (41 – 48)	42 (39,5 – 45)	0,114
EDFPPVE (mm)	6,4 ± 1,0	6,3 ± 1,6	0,674
IMVE (gr/m^2.7^)	68,9 ± 13,7	62,9 ± 12	0,039
FE (%)	68,9 ± 13,7	62,9 ± 12	0,171
E (cm/s)	98,95 (88,8 – 114)	95 (80,75 – 100)	0,166
A (cm/s)	54,35 (43,92 – 72,6)	48 (40,85 – 57,3)	0,027
Ea (cm/s)	12,1 ± 2,2	11,8 ± 1,9	0,627
Aa (cm/s)	5,8 ± 1,2	6,1 ± 1,4	0,383
Sa (cm/s)	7,5 ± 1,0	7,2 ± 1,0	0,210
E/Eas	8,32 (6,49 – 10,56)	7,91 (6,93 – 8,89)	0,261
TCIV	54,1 ± 7,6	47,6 ± 7,1	<0,001
TRIV	55,9 ± 9,1	46,9 ± 8,3	<0,001
TE	282,5 ± 23,6	283,2 ± 22,5	0,889
IDM	0,38 ± 0,05	0,33 ± 0,04	<0,001

*DSIV: diâmetro do septo interventricular; DDFVE: diâmetro diastólico final do ventrículo esquerdo; EDFPPVE: espessura diastólica final da parede posterior do ventrículo esquerdo; IMVE: índice de massa do ventrículo esquerdo; TCIV: tempo de contração isovolumétrica; TRIV: tempo de relaxamento isovolumétrico; TE: tempo de ejeção; IDM: índice de desempenho miocárdico. Variáveis contínuas com distribuição normal são expressas como média ± desvio padrão, aquelas com distribuição não normal como mediana (intervalo interquartil)* .

**Tabela 3:** Características da válvula aórtica, tamanho da aorta e parâmetros de elasticidade

Variável	Pacientes (n = 44)	Controles (n = 41)	p-valor
Anel (mm/m^2^)	14,03 (12,75 – 16,87)	14,28 (12,51 – 17,02)	0,363
Escore-z do anel	0,06 ± 1,1	-0,24 ± 0,96	0,185
Seio da Valsalva (mm/m^2^)	20,4 ± 4,8	20,5 ± 3,7	0,939
Escore-z do Seio da Valsalva	-0,19 ± 1,3	0,23 ± 0,83	0,08
Junção sinotubular (mm/m^2^)	15,87 (14,19 – 20,07)	15,80 (14,33 – 18,42)	0,239
Escore-z da Junção sinotubular	0,03 ± 1,2	-0,03 ± 0,8	0,76
Aorta ascendente (mm/m^2^)	20,1 ± 5,1	18,2 ± 3	0,04
Escore-z da aorta ascendente	1,37 ± 1,24	0,4 ± 0,9	<0,001
Velocidade aórtica (m/s)	1,6 (1,4 – 1,9)	1,1 (0,95 – 1,25)	<0,001
IR	2,69 (1,81 – 3,35)	2,5 (2,09 – 3,92)	0,529
ID	0,01± 0,004	0,009 ± 0,004	0,736
Distensão	21,7 ± 8,6	21,4 ± 10,2	0,883

*IR: índice de rigidez; ID: índice de distensibilidade. Variáveis contínuas com distribuição normal são expressas como média ± desvio padrão, aquelas com distribuição não normal como mediana (intervalo interquartil)* .

**Tabela 4:** Hemodinâmica periférica e central

Variável	Pacientes (n = 44)	Controles (n = 41)	p-valor
PAS periférica (mmHg)	109,2 ± 7,2	109,2 ± 6	0,996
PAD periférica (mmHg)	64,7 ± 5,5	64,2 ± 5,4	0,675
Frequência cardíaca (b/dk)	79,5 ± 11,5	80,9 ± 12,4	0,578
PP periférica	44,4 ± 6	44,8 ± 5,8	0,759
PAS central	98 (94,25-102)	98 (91 – 101)	0,437
PAD central	66,5 ± 5,8	65,8 ± 5,4	0,588
Alx@75 (%)	18,5 ± 8,3	20,6 ± 8,8	0,256
VOP (m/sec)	4,5 (4,4 – 4,6)	4,5 (4,4 – 4,6)	0,528

*PAS: pressão arterial sistólica; PAD: pressão arterial diastólica; PP: pressão de pulso; Alx @ 75: índice de aumento normalizado para uma frequência cardíaca de 75 batimentos/min; VOP: velocidade da onda de pulso. Variáveis contínuas com distribuição normal são expressas como média ± desvio padrão, aquelas com distribuição não normal como mediana (intervalo interquartil)* .

A análise de correlação revelou que o índice de desempenho miocárdico foi positivamente correlacionado com o diâmetro da aorta ascendente (r = 0,275; p = 0,01), velocidade aórtica (r = 0,501; p <0,001) e velocidade A (r = 0,351, p = 0,001). Além disso, o diâmetro da aorta ascendente foi positivamente correlacionado com o índice de massa ventricular esquerda (r = 0,273, p = 0,02). A análise de regressão linear múltipla revelou uma associação independente entre o índice de desempenho miocárdico e o diâmetro da aorta ascendente (p = 0,01) e a velocidade aórtica (p <0,001). A análise de multicolinearidade também foi realizada, e os valores do fator de influência da variância (FIV) das variáveis independentes foram menores que 5 (FIV = 1,349, 1,467, respectivamente).

## Discussão

A rigidez arterial de crianças com válvula aórtica bicúspide com função valvar preservada não mostrou estar aumentada quando utilizamos o método oscilométrico em nosso estudo. No entanto, diâmetros aumentados de aorta ascendente e funções miocárdicas globais diminuídas foram detectados nessas crianças, em comparação com seus pares saudáveis.

Como observado em pacientes com válvula aórtica bicúspide, a aortopatia caracterizada por dilatação da aorta ascendente e aumento da rigidez arterial constitui um risco de dissecção aórtica que ocorre frequentemente durante a idade adulta. ^30^ Utilizando a análise da onda de pulso baseada na tonometria de aplanação, Shim et al., ^7^ revelaram que a aorta central é mais rígida em pacientes adultos com válvula aórtica bicúspide. ^7^ De maneira similar, Wang et al., ^5^ relataram menor vasodilatação mediada por fluxo relacionada ao tamanho aumentado e propriedades elásticas diminuídas da aorta ascendente em adultos com válvula aórtica bicúspide sem disfunção valvar significativa. ^5^ Em estudos que investigaram as propriedades da elasticidade aórtica em pacientes pediátricos, as medidas ultrassonográficas são utilizadas de maneira prevalente para identificar as características de elasticidade da aorta ascendente. ^11^^,^^12^^,^^16^ Erroz et al. relataram maior índice de rigidez aórtica e menor *strain* e distensibilidade aórtica em crianças com válvula aórtica bicúspide isolada de maneira consistente com aumento da rigidez arterial e diminuição da elasticidade. ^11^ Da mesma forma, Ekici et al., ^12^ e Weisman et al., ^16^ relataram propriedades elásticas diminuídas da aorta ascendente em crianças com válvula aórtica bicúspide funcional. Em contraste com esses estudos, que se concentraram apenas na aorta ascendente, o estudo de Eroğlu et al., ^31^ avaliou também a aorta torácica descendente e relatou que a aorta ascendente em crianças com válvula aórtica bicúspide é mais distensível e menos rígida em comparação com seus pares saudáveis. Eles também relataram que não houve diferença entre os grupos em termos de elasticidade em idades mais avançadas. No mesmo estudo, o nível de rigidez arterial medido por vasodilatação mediada por fluxo foi relatado como sendo similar entre pacientes e pares saudáveis em todas as faixas etárias. No presente estudo, em concordância com a literatura, as crianças com válvula aórtica bicúspide apresentaram aortas ascendentes mais largas do que seus pares saudáveis. Esse achado confirma que a aortopatia começa na infância, independentemente das funções valvares. Entretanto, similar aos achados de Eroğlu et al., ^31^ os pacientes pediátricos tinham características semelhantes aos seus pares saudáveis em termos de elasticidade da aorta ascendente, e não houve diferença entre os grupos quanto ao nível de rigidez arterial com base nos resultados da análise oscilométrica da onda de pulso, que é descrito como o método mais objetivo e confiável em nosso estudo. O motivo da ausência de diferença significativa em termos de elasticidade e rigidez arterial pode estar relacionado à ausência de pacientes com dilatação aórtica significativa. No entanto, observou-se que a dilatação da aorta apresentava-se em nível moderado, pois não havia nenhum paciente com escore-z da aorta ascendente >4. Por outro lado, a maioria dos cardiologistas pediátricos inicia o uso de medicamentos para retardar a dilatação aórtica e diminuir o risco de dissecção. ^32^ Esses medicamentos que têm efeitos positivos no remodelamento vascular e miocárdica, diminuem a rigidez arterial. ^33^ O presente estudo não incluiu pacientes com medicação preventiva; portanto, nossos resultados são mais confiáveis.

No presente estudo, como indicador global das funções miocárdicas do ventrículo esquerdo, níveis elevados de índice de desempenho miocárdico foram causados por tempos de contração isovolumétrica e de relaxamento isovolumétrico significativamente prolongados. Isso sugeriu que as funções sistólica e diastólica foram afetadas subclinicamente em crianças com uma válvula aórtica bicúspide funcional. Além disso, os níveis significativamente elevados da velocidade A indicaram alteração nas funções diastólicas do ventrículo esquerdo. As causas potenciais de remodelamento miocárdico identificadas em pacientes com válvula aórtica bicúspide têm sido explicadas como carga aumentada causada por estenose e/ou disfunção da válvula aórtica e carga sistólica miocárdica concomitante, causada por rigidez arterial, a qual estava aumentada em comparação com controles saudáveis. ^6^ O presente estudo não incluiu casos com anormalidades valvares funcionais hemodinamicamente significativas, presentes em um nível que possa afetar a estrutura miocárdica, o diâmetro aórtico ou as funções arteriais, bem como o fato de que os níveis de rigidez arterial dos pacientes foram determinados para serem semelhantes aos de seus pares saudáveis. Portanto, independentemente das funções valvares aórticas e da rigidez arterial, as características comuns na etiologia da aortopatia também podem desempenhar um papel no remodelamento ventricular esquerda. A associação entre os tamanhos da aorta ascendente e o índice de desempenho miocárdico apoia a presença de alterações histopatológicas comuns. Assim, a dilatação aórtica em pacientes com válvula aórtica bicúspide tem sido associada a níveis mais baixos de óxido nítrico endotelial, degeneração das fibras elásticas, apoptose de células musculares lisas, remodelamento extracelular anormal e necrose cística medial aórtica, ao invés de fatores hemodinâmicos. ^5^^,^^8^^,^^31^ Em estudos anteriores, em concordância com os achados do presente estudo, as alterações histopatológicas que afetam a ocorrência de dilatação aórtica também podem ter desempenhado um papel no remodelamento miocárdico identificado em pacientes com válvula aórtica bicúspide com as funções valvares preservadas. ^6^

### Limitações e pontos fortes

A tonometria de aplanação é o método mais comumente utilizado para medir a rigidez arterial. Não realizamos a tonometria de aplanação devido às suas limitações de uso em crianças, como manter um sinal suficientemente forte, cooperação e variabilidade da frequência cardíaca. Os estudos sobre a validação do método oscilométrico para crianças são limitados na literatura; entretanto, foi relatado que é um método de fácil utilização e confiável para avaliação dos parâmetros de rigidez arterial. ^14^ Além disso, os parâmetros de elasticidade aórtica amplamente utilizados em outros estudos para avaliação da rigidez arterial em crianças com válvula aórtica bicúspide também foram utilizados em nosso estudo. Outra limitação do nosso estudo é que pacientes com dilatação aórtica acentuada não foram incluídos, em decorrência da exclusão de pacientes com medicação preventiva. Assim, o efeito confundidor da medicação foi excluído.

## Conclusão

A válvula aórtica bicúspide é uma doença que não se limita apenas à válvula aórtica e onde se inicia o remodelamento da aorta ascendente e do miocárdio do ventrículo esquerdo na infância. O tamanho da aorta ascendente pode ser mais preditivo do que as funções valvares no remodelamento miocárdico. A rigidez arterial, que desempenha um papel importante no surgimento de complicações aórticas em pacientes com válvula aórtica bicúspide, pode não ser um fator de risco grave em pacientes pediátricos sem dilatação acentuada da aorta ascendente. Embora o método oscilométrico pareça ser confiável na avaliação da rigidez arterial em crianças com válvula aórtica bicúspide isolada, mais estudos abrangentes sobre esse assunto são necessários.

## References

[1] Ward C. Clinical significance of the bicuspid aortic valve. Heart. 2000;83(1):81-5. 10.1136/heart.83.1.81 PMC1729267 10618341

[2] Michelena HI, Prakash SK, Della Corte A, Bissell MM, Anavekar N, Mathieu P, et al. Bicuspid aortic valve: identifying knowledge gaps and rising to the challenge from the International Bicuspid Aortic Valve Consortium (BAVCon). *Circulation.* 2014;129(25):2691-704. 10.1161/CIRCULATIONAHA.113.007851 PMC4145814 24958752

[3] Mordi I, Tzemos N. Bicuspid aortic valve disease: a comprehensive review. *Cardiol Res Pract.* 2012;2012:196037. https://doi:10.1155/2012/196037. 10.1155/2012/196037 PMC3368178 22685681

[4] Tadros TM, Klein MD, Shapira OM. Ascending aorta dilatation associated with bicuspid aortic valve. Pathophsiology, molecular biology, and clinical implications. *Circulation.* 2009;119(6):880-90. 10.1161/CIRCULATIONAHA.108.795401 19221231

[5] Wang YB, Li Y, Deng YB, Zhang J, Sun J, Zhu Y, Li L, et al. Enlarged size and impaired elastic properties of the ascending aorta are associated with endothelial dysfunction and elevated plasma Matrix Metalloproteinase-2 level in patients with bicuspid aortic valve. *Ultrasound Med Bio.l* 2018;44(5):955-62. 10.1016/j.ultrasmedbio.2018.01.012 29472114

[6] Li Y, Deng YB, Bi XJ, Liu YN, Zhang J, Li L, et al. Evaluation of myocardial strain and aortic elasticity in patients with bicuspid aortic valve. *J Huazhong Univ Sci Technolog* Med Sci .2016;36(5):747-51. 10.1007/s11596-016-1656-x 27752907

[7] Shim CY, Cho IJ, Yang WI, Kang MK, Park S, Ha JW, et al. Central aortic stiffness and its association with ascending aorta dilation in subjects with a bicuspid aortic valve. *J Am Soc Echocardiogr.* 2011;24(8):847-52. 10.1016/j.echo.2011.04.017 21652174

[8] Aicher D, Urbich C, Zeiher A, Dimmeler S, Schäfers HJ. Endothelial nitric oxide synthase in bicuspid aortic valve disease. *Ann Thorac Surg.* 2007;83(4):1290-4. 10.1016/j.athoracsur.2006.11.086 17383329

[9] Ahmadizar F, Voortman T. Arterial stiffness in childhood: A predictor for later cardiovascular disease; Structural abnormalities of great arterial walls in congenital heart disease: Light and electron microscopic analyses. *Eur J Prev Cardiol.* 2018;25(1):100-2. 10.1177/2047487317743046 PMC5724587 29154683

[10] Niwa K, Perloff JK, Bhuta SM, Laks H, Drinkwater DC, Child JS, et al. Structural abnormalities of great arterial walls in congenital heart disease: Light and electron microscopic analyses. *Circulation.* 2001;103(3): 393–400. 10.1161/01.cir.103.3.393 11157691

[11] Oulego-Erroz I, Alonso-Quintela P, Mora-Matilla M, Gautreaux Minaya S, Lapeña-López de Armentia S. Ascending aorta elasticity in children with isolated bicuspid aortic valve. *Int J Cardiol.* 2013168(2):1143-6. 10.1016/j.ijcard.2012.11.080 23232455

[12] Ekici F, Uslu D, Bozkurt S. Elasticity of ascending aorta and left ventricular myocardial functions in children with bicuspid aortic valve. *Echocardiography.* 2017;34(11):1660-6. 10.1111/echo.13664 28833432

[13] Laurent S, Cockcroft J, Van Bortel L, Boutouyrie P, Giannattasio C, Hayoz D, et al. Expert consensus document on arterial stiffness: methodological issues and clinical applications. *Eur Heart J.* 2006;27(21):2588–605. 10.1093/eurheartj/ehl254 17000623

[14] Vanderschuren MM, Uiterwaal CS, van der Ent CK, Eising JB. Feasibility and characteristics of arterial stiffness measurement in preschool children *. Eur J Prev Cardiol* .2017;24-17)1895-902. 10.1177/2047487317721979 PMC5680906 28728487

[15] Tokgöz S, Yılmaz D, Tokgöz Y, Çelik B, Bulut Y. The evaluation of arterial stiffness of essential hypertension and white coat hypertension in children: A case-control study. *Cardiol Young.* 2018;28(3):403-8 10.1017/S1047951117002025 29223189

[16] Weismann CG, Lombardi KC, Grell BS, Northrup V, Sugeng L. Aortic stiffness and left ventricular diastolic function in children with well-functioning bicuspid aortic valves. *Eur Heart J Cardiovasc Imaging.* 2016;17(2):225–30. 10.1093/ehjci/jev151 PMC4882883 26072912

[17] Baumgartner H, Hung J, Bermejo J, Chambers JB, Evangelista A, Griffin BP, et al. American Society of Echocardiography; European Association of Echocardiography. Echocardiographic assessment of valve stenosis: EAE/ASE recommendations for clinical practice. *J Am Soc Echocardiogr* .2009;22(1):1-23. 10.1016/j.echo.2008.11.029 19130998

[18] Zoghbi WA, Adams D, Bonow RO, Enriquez-Sarano M, Foster E, Grayburn PA, et al. Recommendations for noninvasive evaluation of native valvular regurgitation: A Report from the American Society of Echocardiography developed in collaboration with the Society for Cardiovascular Magnetic Resonance. *J Am Soc Echocardiogr.* 2017;30(4):303-.71. 10.1016/j.echo.2017.01.007 28314623

[19] S.A. Goldstein, A. Evangelista, S. Abbara, Arai A, Asch FM, Badano LP, et al. Multimodality imaging of diseases of the thoracic aorta in adults: from the American Society of Echocardiography and the European Association of Cardiovascular Imaging: endorsed by the Society of Cardiovascular Computed Tomography and Society for Cardiovascular Magnetic Resonance. *J Am Soc Echocardiogr* 2015;28(2):119-82. 10.1016/j.echo.2014.11.015 25623219

[20] Lopez L, Colan SD, Frommelt PC, Ensing GJ, Kendall K, Younoszai AK, et al. Recommendations for quantification methods during the performance of a pediatric echocardiogram: a report from the Pediatric Measurements Writing Group of the American Society of Echocardiography Pediatric and Congenital Heart Disease Council. *J Am Soc Echocardiogr.* 2010;23(5):465–95. 10.1016/j.echo.2010.03.019 20451803

[21] Devereux RB, Alonso DR, Lutas EM, Gottlieb GJ, Campo E, Sachs I, et al. Echocardiographic assessment of left ventricular hypertrophy: comparison to necropsy findings. *Am J Cardiol* 1986;57(6):450-8. 10.1016/0002-9149(86)90771-x 2936235

[22] Chengode S. Left ventricular global systolic function assessment by echocardiography. *Ann Card Anaesth.* 2016;19(Supplement):S26–S34. 10.4103/0971-9784.192617 PMC5100240 27762246

[23] Tei C., Ling L.H., Hodge D.O. New index of combined systolic and diastolic myocardial performance: a simple and reproducible measure of cardiac function—a study in normals and dilated cardiomyopathy. *J Cardiol.* 1995;26(6):357–66. 8558414

[24] Fahey M, Ko HH, Srivastava S, Lai WW, Chatterjee S, Parness IA, et al. A comparison of echocardiographic techniques in determination of arterial elasticity in the pediatric population. *Echocardiography* 2009;26(5):567–73. 10.1111/j.1540-8175.2008.00849.x 19452610

[25] Stoner L, Lambrick DM, Westrupp N, Young J, Faulkner J. Validation of oscillometric pulse wave analysis measurements in children. *Am J Hypertens.* 2014;27(6):865-72. 10.1093/ajh/hpt243 24390294

[26] Weber T, Wassertheurer S, Rammer M, Maurer E, Hametner B, Mayer CC, et al. Validation of a brachial cuff-based method for estimating central systolic blood pressure *. Hypertension* 2011;58(5):825–32. 10.1161/HYPERTENSIONAHA.111.176313 21911710

[27] Elmenhorst J, Weberruss H, Mayr M, Pfister K, Oberhoffer R. Comparison of two measurement devices for pulse wave velocity in children: which tool is useful to detect vascular alterations caused by overweight? *Front Pediatr.* 2019;7:334. 10.3389/fped.2019.00334 PMC6710322 31482076

[28] Solanki JD, Munshi HB, Mehta HB, Shah CJ. Central hemodynamics and arterial stiffness in Gujarati diabetics not receiving any antihypertensive: A case-control study based on oscillometric pulse wave analysis. *J Family Med Prim Care.* 2019 Aug 20;8:1352–8. 10.4103/jfmpc.jfmpc_117_19 PMC6510094 31143720

[29] Warner PJ, Al-Quthami A, Brooks EL, Kelley-Hedgepeth A, Patvardhan E, Kuvin JT, et al. Augmentation index and aortic stiffness in bicuspid aortic valve patients with non-dilated proximal aortas. *BMC Cardiovasc Disord.* 2013 Mar 15;13:19. https://doi:10.1186/1471-2261-13-19. 10.1186/1471-2261-13-19 PMC3602003 23496804

[30] Pepe G, Nistri S, Giusti B, Sticchi E, Attanasio M, Porciani C, et al. Identification of Fibrillin 1 gene mutations in patients with bicuspid aortic valve (BAV) without Marfan syndrome. *BMC Med Genet.* 2014;15:23. 10.1186/1471-2350-15-23 PMC3937520 24564502

[31] Eroğlu E, Akalın F, Çetiner N, Şaylan BÇ. Aortic elasticity and the influence of valve morphology in children with bicuspid aortic valve. *Cardiol Young.* 2018;28(11):1338-44. 10.1017/S1047951118001348 30079852

[32] Hussain A, Warren A.E, Chen RPC, Dhillon SS. Practice variation among Canadian pediatric cardiologists in medical management of dilated ascending aorta in patients with bicuspid aortic valve. *CJC Open.* 2019;1(3)119-22. 10.1016/j.cjco.2019.03.002 PMC7063647 32159094

[33] Shahin Y, Khan J.A, Chetter I. Angiotensin converting enzyme inhibitors effect on arterial stiffness and wave reflections: a meta-analysis and meta-regression of randomised controlled trials. *Atherosclerosis.* 2012;221(1):18-33. 10.1016/j.atherosclerosis.2011.12.005 22209214

