# Supplementary material for: Rigidez Arterial e Funções Miocárdicas do Ventrículo Esquerdo em Crianças com Válvula Aórtica Bicúspide Funcional
Source: Arq Bras Cardiol. 2021 Sep 3;117(6):1126–33. [Article in Portuguese] doi: 10.36660/abc.20200657 (PMC8757143; doi:10.36660/abc.20200657)
Supplement: Translation

# Translation: Arterial Stiffness and Left Ventricular Myocardial Function in Children with a Well-Functioning Bicuspid Aortic Valve

**Authors:** Pelin Kosger, Tugcem Akin, Hikmet Kiztanir, Birsen Ucar

**Affiliations:** 1 Eskisehir Turkey Eskisehir Osmangazi University, Faculty of Medicine, Department of Pediatric Cardiology, Eskisehir - Turkey; 2 Eskisehir Turkey Eskisehir State Hospital, Pediatric Cardiology Clinic, Eskisehir - Turkey

**Keywords:** Aortic Stiffness, Dilatation Pathologic, Ventricular Function, Left, Pulse Wave Analysis, Myocardial, Child

## Abstract

**Background:**

Arterial stiffness is an important predictor factor of aortopathy and myocardial remodeling in patients with a bicuspid aortic valve and it might be increased in childhood.

**Objective:**

To assess the arterial stiffness and left ventricular myocardial function in children with a well-functioning bicuspid aortic valve.

**Methods:**

Forty-four children with a bicuspid aortic valve and 41 healthy peers with a tricuspid aortic valve were included in this case-control study. Diameters and the related z-scores of the aortic root and ascending aorta were obtained. As for the left ventricular myocardial function, along with the mitral inflow velocities and M-Mode parameters, myocardial velocities and time intervals were assessed with tissue Doppler imaging. A pulse wave analysis was performed by oscillometric device (Mobil-o-Graph). A p value <0.05 was considered significant.

**Results:**

The left ventricular mass index, mitral inflow A velocity, diameter and z-score of the ascending aorta, and myocardial performance index were significantly higher in patients (p=0.04, p=0.02,p=0.04, p<0.001,and p<0.001 respectively). The myocardial performance index was positively correlated with the diameter of the ascending aorta and A velocity (r=0.272;p=0.01, r=356;p=0.001, respectively). The multivariate analysis revealed that the myocardial performance index was related to the ascending aorta diameter (p=0.01). The augmentation index and pulse wave velocity were similar between the groups (p>0.05).

**Conclusion:**

According to the oscillometric pulse wave analysis, the children with a well-functioning bicuspid aortic valve had similar arterial stiffness to that of the healthy peers. The ascending aorta diameter was established as an independent predictor of left ventricular myocardial function. Arterial stiffness may not be a severe risk factor in pediatric patients without marked ascending aorta dilation.

## Introduction

A bicuspid aortic valve is the most common congenital cardiac malformation and occurs in 1-2% of the general population. ^1^ In addition to impaired valvular function, individuals are also at risk of aortopathy, which may result in aortic dissection and aneurysm formation. ^2^^,^^3^ There is no direct association between valvular dysfunction and aortopathy. ^4^ The presence of aorta dilation, even in patients with normal valvular function, may be related to a different pathophysiology of aortopathy. ^5^ In comparison with healthy controls, patients with bicuspid aortic valve without apparent valvular dysfunction have shown decreased aortic elasticity and increased central aortic stiffness. ^6^ Furthermore, in these individuals, central aortic stiffness measured using the ambulatory pulse wave velocity monitoring method is positively correlated with the degree of aortic dilation. ^7^ Therefore, vascular remodeling has been identified as the primary cause of arterial stiffness that impacts the monitoring process in these patients. ^8^ The level of arterial stiffness, a predictor of the course of cardiovascular diseases, tends to increase with age. However, examination of intraoperative biopsy and necropsy samples have shown that in many cases of congenital heart diseases, arterial stiffness increases as from childhood. ^9^^,^^10^ Patients with a bicuspid aortic valve show progressive aortic dilation during their childhood, and data regarding aortic elasticity and arterial stiffness are based on ultrasonographic methods, which are highly limited in number. ^11^^,^^12^ The gold standard method of arterial stiffness measurement is pulse wave analysis and the most commonly pulse wave analysis technique is tonometry; however, this technique can be time-consuming and challenging, especially when used in young children. ^13^ Oscillometric devices are user-friendly, practical for use in the clinical setting, and a reliable method for evaluating central blood pressure and arterial stiffness parameters, even in children. ^14^^,^^15^

The patients with a bicuspid aortic valve also suffer from myocardial remodeling, regardless of valvular functions and aortopathy. However, to confirm that myocardial remodeling is not only associated with valvular functions, studies including cases with intact valve function without additional risk factors are required. Pediatric cases comprise an ideal group of patients for this purpose, and currently there are only a few studies in the literature reporting that the left ventricular diastolic functions are affected in pediatric patients. ^12^^,^^16^ We aimed to evaluate arterial stiffness using the oscillometric method and to determine whether it is compatible with the level of ultrasonographic arterial elasticity in children with a well-functioning bicuspid aortic valve. Furthermore, it is aimed to assess the global myocardial function via tissue Doppler imaging-derived myocardial performance index in our study.

## Materials and Methods

### Study population

A total of 44 patients (7-18 years of age) followed in a pediatric cardiology clinic with a diagnosis of bicuspid aortic valve were enrolled in the study. These patients had no apparent aortic valvular dysfunction, neither had they received preventive medication for aortopathy. Forty-one children who had healthy tricuspid aortic valves and with similar demographic and anthropometric characteristics were included as the control group. Patients with moderate to severe valve insufficiency, with a valve velocity >2m/s, who had undergone prior surgery or percutaneous intervention, and had an additional repaired or unrepaired heart disease (e.g., coarctation of the aorta), as well as those with a body mass index and systolic blood pressure of >95^th^percentile were excluded from the study. Moreover, patients who had received preventive medication were not included, aiming to avoid the confounding effects of medications on myocardial mechanics and arterial stiffness. As demonstrated by laboratory tests, none of the children in the study population had hypercholesterolemia. Patients gave a written informed consent to participate in the study and the study was approved by the local Ethics Committee of Eskisehir Osmangazi University on April 04, 2018.

### Echocardiography

Transthoracic echocardiography was performed by a single experienced pediatric cardiologist using the commercially available equipment Affinity 70 (Philips Medical Systems, Bothell, WA, USA) with 2–4 and 4–8 MHz broadband probes. The vascular examination was performed using VIVID I color Doppler ultrasonography (General Electric Ultrasound Systems, Mountain View, CA, USA) equipment with a 12-MHz linear probe.

#### Assessment of aortic valve morphology and functions

Aortic valve morphology was evaluated on the parasternal long and short axis sections. Probe frequency was selected according to patient size. A definitive bicuspid aortic valve was diagnosed when only two valve leaflets were unequivocally identified at systole and diastole, with a clear fish-mouth appearance in systole. Leaflet phenotype was defined as anteroposterior (left and right cusp fusion) or right–left (right and noncoronary cusp fusion). For aortic valve stenosis evaluation, peak aortic velocity was measured using continuous wave Doppler while the cursor was maintained at the level of the Valsalva sinus in the five-chamber view, and a value of >2.5 m/s was considered indicative of aortic stenosis. ^17^ To determine the degree of aortic valve insufficiency, we used the proportion of the aorta insufficiency diameter measured on the color Doppler images, which were obtained from the long-axis section, in relation to the left ventricular outflow tract diameter. Ratios <25%, 25-64%, and ≥65% generally indicate mild, moderate, and severe aortic regurgitation, respectively. ^18^

#### Measurement of aortic root and ascending aorta diameter

Measurements of the four aortic segments, including the aortic annulus, Valsalva sinus, sinotubular junction, and proximal ascending aorta 2 cm above the sinotubular junction, were obtained in the parasternal long-axis view at the end diastole, leading-edge-to-leading-edge, and perpendicular to the long axis of the aorta. ^19^ Aortic dimensions were normalized to the body surface area. A z-score of >2 was considered abnormal.

#### Left ventricular M-mode and tissue Doppler echocardiography

Left ventricular internal dimensions, interventricular septum thickness, and posterior wall thickness were measured at the end diastole using two-dimensional M-mode echocardiography according to the pediatric guidelines of the American Society of Echocardiography. ^20^ Left ventricular mass was calculated according to the Devereux Formula ^21^ and indexed to the height. Left ventricular ejection fraction was calculated using the Teichholz formula. ^22^ The Doppler sample volume was placed at the tips of mitral leaflets to obtain the left ventricular inflow waveforms from the apical four-chamber view. The mitral inflow early diastolic velocity (E) and the late diastolic velocity (A) were also measured. To measure the longitudinal myocardial velocities, the sample volume was placed on the septal corner of the mitral annulus to obtain waveforms from the apical four-chamber view. Early diastolic mitral annular velocity (Ea) and late diastolic mitral annular velocity (Aa) were measured, and their ratio (Ea/Aa) was calculated to estimate left ventricular filling pressure. Cardiac time intervals, including the isovolumic relaxation time, isovolumic contraction time, and ejection time, were obtained by tissue Doppler imaging, and the myocardial performance index calculated according to Tei’s formula: myocardial performance index = (isovolumic contraction time + isovolumic relaxation time)/ejection time. ^23^ For each quantitative parameter, three consecutive beats were averaged.

#### Assessment of aortic elasticity

Aortic elasticity was assessed using the two-dimensional guided M-mode evaluation of systolic and diastolic aortic diameters, 2 cm above the aortic valve; diastolic diameter was obtained at the R wave peak at the simultaneously recorded electrocardiogram, whereas systolic diameter was measured at the maximal anterior motion of the aortic wall.

Aortic strain, aortic distensibility, and aortic stiffness index were calculated using the formulas below:

*Aortic strain* = 100 (Systolic diameter-Diastolic diameter) / Diastolic diameter

*Aortic stiffness index* = natural logarithm (Systolic blood pressure/Diastolic blood pressure) / [(Systolic diameter-Diastolic diameter/Diastolic diameter]

*Aortic distensibility* (cm^2^/dyn/10^-6^) = 2x (Systolic diameter-Diastolic diameter) / [(Systolic blood pressure – Diastolic blood pressure) X Diastolic diameter] ^24^

### Arterial stiffness measurement

For pulse wave analysis and blood pressure monitoring, the Mobil-O-Graph (IEM, Industrielle Entwicklung Medizintechnik und Vertriebsgesellschaft mbH, Stolberg, Germany) device and the ARCSolver pulse wave analysis software (AIT Austrian Institute of Technology GmbH, Vienna, Austria) were used. 24-hour blood pressure monitoring was performed by connecting a cuff of an appropriate size for upper arm circumference. During the test, the peripheral and central systolic blood pressure, peripheral and central diastolic blood pressure, pulse, pulse wave velocity and augmentation index were measured.

The Mobil-O-Graph is an oscillometric ambulatory blood pressure measurement device that is appropriate for use in children. ^25^^,^^26^ After blood pressure measurement, the cuff is inflated to the brachial diastolic pressure level and the oscillations (pulse waves) are recorded for 10 seconds. After the 24-hour measurement circle, all measurements are transferred to the HMS client software and analyzed using the ARCSolver software, which has been applied to children. ^27^

Aortic pulse wave velocity and augmentation index at a heart rate (HR) of 75 beats/minute (AIx@75) are markers of arterial stiffness. ^28^ Aortic pulse wave velocity is the speed at which pulse waves travel in the aortic wall and is a central arterial stiffness measure. AIx@75 is derived from the augmentation pressure and pulse pressure of a pulse wave. Pulse wave is a summation of forward (producing first systolic peak) and reflected (producing second peak) waves. Increase in the pulse wave amplitude due to pulse wave reflection is known as pulse augmentation, and its contribution to pulse wave amplitude is known as augmentation pressure. Moreover, the percentage of pulse wave amplitude due to augmentation pressure is known as augmentation index, which is dependent on heart rate. Mobil-o-Graph provides it at heart rate 75, a measure of peripheral arterial stiffness. ^29^

### Statistical Analysis

Statistical analysis was performed using the Statistical Package for Social Sciences, version 18 (SPSS, Chicago, USA). The sample size was determined by the G-power analysis software with a statistical power of 85%. The Kolmogorov-Smirnov test was used for the assessment of normal distribution. The results of continuous variables were expressed as mean ± standard deviation (SD) or median (percentile 25 and 75, interquartile range, IQR). The groups were compared using independent samples’ T test for continuous variables, and the Mann–Whitney U test was used for non-normally distributed variables. The Chi-square test was used for the gender comparison between the groups. Spearman’s correlation test was used for correlations. Multiple linear regression analysis using the Backward method was performed to assess the independent predictor of the myocardial performance index in patients with bicuspid aortic valve. The statistical significance level was set at p <0.05.

## Results

The median age of the entire study population (n = 85) was 12 (IQR=8.5–14) and ranged from 7 to 18 years. Twelve patients in the bicuspid aortic valve group (12/44) and 18 cases in the control group (18/41) were females (p=0.084). There was no significant difference in terms of age, weight, height, body surface area, body mass index, serum lipid profile, and glucose levels between the groups ( Table 1 ).

**Table 1:** Baseline demographic, anthropometric and clinical characteristics of the groups

Variable	Patients (n = 44)	Controls (n = 41)	p-value
Age (years)	12 (9 - 15)	12 (8 -14)	0.609
Gender (female,%)	12 (27.27)	18 (43.90)	0.084
Height (cm)	147.8 ± 20.1	146.8 ± 15.5	0.798
Weight (kg)	42.3 ± 16.4	38.6 ± 14	0.268
BMI (kg/m^2^)	18 ± 0.3	17 ± 0.3	0.088
TC (mg/dl)	141.1 ± 23.1	148.3 ± 28.4	0.308
TG (mg/dl)	91.6 ± 38.9	86.6 ± 31.9	0.113

*BMI: Body mass index; TC: total cholesterol; TG: total triglycerides. Continuous variables with normal distribution are expressed as mean ± standard deviation, and those with non-normally distribution as median (interquartile range)* .

Echocardiographic and aortic elasticity parameters are summarized in Tables 2 and 3 . Mitral inflow A velocity, isovolumetric relaxation time, isovolumetric contraction time, and myocardial performance index were significantly higher in patients than in controls (p = 0.03, <0.001, <0.001, <0.001, respectively). Fusion of the left and right coronary cusps defined as the anteroposterior phenotype was the predominant phenotype (63.6%). Along with the aortic velocity, the ascending aorta diameter and z-score was higher in patients (p < 0.001, p = 0.04, p < 0.001, respectively). All aortic elasticity parameters were similar between the groups. The central and peripheral hemodynamic variables, Alx@75 and pulse wave velocity values, which showed no significant difference between the groups, are shown in Table 4 .

**Table 2:** Echocardiographic measurements in patients and controls

Variable	Patients (n = 44)	Controls (n = 41)	p-value
IVSDd (mm)	6.9 ± 1.0	6.7 ± 0.9	0.429
LVEDd (mm)	45 (41 – 48)	42 (39.5 – 45)	0.114
LVPWDd (mm)	6.4 ± 1.0	6.3 ± 1.6	0.674
LVMI (gr/m^2.7^)	68.9 ± 13.7	62.9 ± 12	0.039
EF (%)	68.9 ± 13.7	62.9 ± 12	0.171
E (cm/s)	98.95 (88.8 – 114)	95 (80.75 – 100)	0.166
A (cm/s)	54.35 (43.92 – 72.6)	48 (40.85 – 57.3)	0.027
Ea (cm/s)	12.1 ± 2.2	11.8 ± 1.9	0.627
Aa (cm/s)	5.8 ± 1.2	6.1 ± 1.4	0.383
Sa (cm/s)	7.5 ± 1.0	7.2 ± 1.0	0.210
E/Ea	8.32 (6.49 – 10.56)	7.91 (6.93 – 8.89)	0.261
IVCT	54.1 ± 7.6	47.6 ± 7.1	<0.001
IVRT	55.9 ± 9.1	46.9 ± 8.3	<0.001
ET	282.5 ± 23.6	283.2 ± 22.5	0.889
MPI	0.38 ± 0.05	0.33 ± 0.04	<0.001

*IVSDd: Interventricular septum diameter; LVEDd: end-diastolic left ventricular internal diameters; LVPWDd: end-diastolic left ventricular posterior wall thickness; LVMI: left ventricular mass index; IVCT: isovolumetric contraction time; IVRT: isovolumetric relaxation time; ET: ejection time; MPI: myocardial performance index. Continuous variables with normal distribution are expressed as mean ± standard deviation, those with non-normally distribution as median (interquartile range)* .

**Table 3:** Aortic valve characteristics, aortic size and elasticity parameters

Variable	Patients (n = 44)	Controls (n = 41)	p-value
Anulus (mm/m^2^)	14.03 (12.75 – 16.87)	14.28 (12.51 – 17.02)	0.363
Anulus z-score	0.06 ± 1.1	-0.24 ± 0.96	0.185
Sinus of Valsalva (mm/m^2^)	20.4 ± 4.8	20.5 ± 3.7	0.939
Sinus of Valsalva z-score	-0.19 ± 1.3	0.23 ± 0.83	0.08
Sinotubular junction (mm/m^2^)	15.87 (14.19 – 20.07)	15.80 (14.33 – 18.42)	0.239
Sinotubular junction z-score	0.03 ± 1.2	-0.03 ± 0.8	0.76
Ascending aorta (mm/m^2^)	20.1 ± 5.1	18.2 ± 3	0.04
Ascending aorta z- score	1.37 ± 1.24	0.4 ± 0.9	<0.001
Aortic velocity (m/s)	1.6 (1.4 – 1.9)	1.1 (0.95 – 1.25)	<0.001
SI	2.69 (1.81 – 3.35)	2.5 (2.09 – 3.92)	0.529
DI	0.01± 0.004	0.009 ± 0.004	0.736
Strain	21.7 ± 8.6	21.4 ± 10.2	0.883

*SI: Stiffness index; DI: distensibility index. Continuous variables with normal distribution are expressed as mean ± standard deviation, those with non-normally distribution as median (interquartile range)* .

**Table 4:** Peripheral and central hemodynamics

Variable	Patients (n = 44)	Controls (n = 41)	p-value
Peripheral SBP (mmHg)	109.2 ± 7.2	109.2 ± 6	0.996
Peripheral DBP (mmHg)	64.7 ± 5.5	64.2 ± 5.4	0.675
Heart rate (beats/dk)	79.5 ± 11.5	80.9 ± 12.4	0.578
Peripheral PP	44.4 ± 6	44.8 ± 5.8	0.759
Central SBD	98 (94.25-102)	98 (91 – 101)	0.437
Central DBP	66.5 ± 5.8	65.8 ± 5.4	0.588
Alx@75 (%)	18.5 ± 8.3	20.6 ± 8.8	0.256
PWV (m/sec)	4.5 (4.4 – 4.6)	4.5 (4.4 – 4.6)	0.528

*SBP: systolic blood pressure; DBP: diastolic blood pressure; PP: pulse pressure; Alx@75, augmentation index normalized for a heart rate of 75 beats/min; PWV: pulse wave velocity. Continuous variables with normal distribution are expressed as mean ± standard deviation, those with non-normally distribution as median (interquartile range)* .

The correlation analysis showed that the myocardial performance index was positively correlated with the ascending aorta diameter (r= 0.275; p= 0.01), aortic velocity (r= 0.501; p <0.001) and A velocity (r= 0.351, p = 0.001). Also, the ascending aorta diameter was positively correlated with the left ventricular mass index (r= 0.273, p= 0.02). The multiple linear regression analysis disclosed an independent association between the myocardial performance index and the ascending aorta diameter (p= 0.01) and aortic velocity (p <0.001). A multicollinearity analysis was also performed, and the variance influence factor (VIF) values of independent variables were found to be less than 5 (VIF = 1.349, 1.467, respectively).

## Discussion

Arterial stiffness in children with a bicuspid aortic valve with preserved valvular function was not found to be increased when using the oscillometric method in our study. However, greater ascending aorta diameters and impaired global myocardial functions were detected in these children, when compared to their healthy peers.

As observed in patients with a bicuspid aortic valve, the aortopathy characterized by ascending aorta dilation and increased arterial stiffness, constitutes a risk for aortic dissection, frequently occurring during adulthood. ^30^ Using applanation tonometry-based pulse-wave analysis, Shim et al. ^7^ revealed that the central aorta is stiffer in adult patients with a bicuspid aortic valve. ^7^ Similarly, Wang et al. ^5^ reported lower flow-mediated vasodilation related to the enlarged size and impaired elastic properties of the ascending aorta in adults with a bicuspid aortic valve without significant valvular dysfunction. ^5^ In studies investigating the properties of aortic elasticity in pediatric patients, ultrasonographic measurements are prevalently used to identify the elasticity characteristics of the ascending aorta. ^11^^,^^12^^,^^16^ Erroz et al. reported higher aortic stiffness index and lower aortic strain and distensibility in children with an isolated bicuspid aortic valve in a manner consistent with increased arterial stiffness and decreased elasticity. ^11^ Similarly, Ekici et al. ^12^ and Weisman et al. ^16^ reported impaired elastic properties of the ascending aorta in children with a well-functioning bicuspid aortic valve. In contrast to these studies, which focused only on the ascending aorta, the study by Eroğlu et al. ^31^ assessed the descending thoracic aorta as well and reported that the ascending aorta in children with a bicuspid aortic valve is more distensible and less stiff compared with that in their healthy peers. They also reported that there was no difference between the groups in terms of elasticity at older ages. In the same study, the level of arterial stiffness measured by flow-mediated vasodilation was reported to be similar between patients and healthy peers in all age groups. In the present study, in agreement with the literature, the children with a bicuspid aortic valve had wider ascending aortas than the healthy peers. This finding supports the fact that aortopathy begins in childhood, regardless of valve function. However, similar to the findings by Eroğlu et al., ^31^ the pediatric patients had similar characteristics as their healthy peers in terms of elasticity of the ascending aorta, and there was no difference between the groups regarding arterial stiffness level based on the oscillometric pulse-wave analysis results, which is described as a more objective and reliable method in our study. The reason for the lack of a significant difference in terms of arterial elasticity and stiffness may be related with the absence of patients with significant aortic dilation. However, aortic dilation was observed to be at a moderate level, as there was no patient with an ascending aorta z-score >4. On the other hand, most pediatric cardiologists start medication to slow down aortic dilation and decrease the risk of dissection. ^32^ These medications that have positive effects on vascular and myocardial remodeling decrease arterial stiffness. ^33^ The present study did not include patients receiving preventive medication, therefore our results are more reliable.

In the present study, as the global indicator of left ventricular myocardial function, a high myocardial performance index was caused by the significantly prolonged isovolumetric contraction time and isovolumetric relaxation time. This suggested that both the systolic and diastolic functions were sub-clinically affected in children with a well-functioning bicuspid aortic valve. In addition, the significantly high levels of A velocity indicated the alteration in the left ventricular diastolic function. The potential causes of myocardial remodeling identified in patients with a bicuspid aortic valve has been explained as an increased load caused by concomitant aortic valve stenosis and/or dysfunction, and myocardial systolic load caused by arterial stiffness, which was found to be increased compared with that in healthy controls. ^6^ The present study does not include cases with hemodynamically significant functional valvular abnormality at a level that might affect the myocardial structure, aortic diameter, or arterial functions, as well as the fact that the arterial stiffness levels in the patients were determined to be similar to those in their healthy peers. Hence, independent from aortic valvular functions and arterial stiffness, the common features in the aortopathy etiology may also play a role in left ventricular remodeling. The association between the ascending aorta sizes and myocardial performance index, support the presence of common histopathological changes. Thus, aortic dilation in patients with a bicuspid aortic valve has been associated with lower endothelial nitric oxide levels, elastic fiber degeneration, smooth muscle cell apoptosis, abnormal extracellular remodeling, and aortic cystic medial necrosis rather than hemodynamic factors. ^5^^,^^8^^,^^31^ In previous studies, in agreement with the present study findings, the histopathological changes that have an effect on the occurrence of aortic dilation might have also played a role in the myocardial remodeling identified in patients with a bicuspid aortic valve in which valvular functions were preserved. ^6^

### Limitations and strengths

Applanation tonometry is the most commonly used method to measure arterial stiffness. We did not perform applanation tonometry because of its use limitations in children, such as maintaining a sufficiently strong signal, cooperation, and heart rate variability. The studies about the validation of the oscillometric method for children are limited in the literature; however, it was reported that it is a user-friendly and reliable method for evaluating arterial stiffness parameters. ^14^ In addition, aortic elasticity parameters, which were widely used for the assessment of arterial stiffness in other studies in children with a bicuspid aortic valve were also performed in our study. Another limitation of our study is the fact that patients with marked aortic dilation were not included, as patients receiving preventive medication were excluded. Thus, the confounding effect of medication was excluded.

## Conclusion

A bicuspid aortic valve is a disease that is not limited to the aortic valve only and where remodeling starts in the ascending aorta and left ventricular myocardium during childhood. The ascending aorta size may be more predictive than valvular functions in myocardial remodeling. Arterial stiffness, which plays an important role in the emergence of aortic complications in patients with a bicuspid aortic valve, may not be a serious risk factor in pediatric patients without marked ascending aorta dilation. Although the oscillometric method seems reliable for the assessment of arterial stiffness in children with an isolated bicuspid aortic valve, further comprehensive studies are needed on this issue.
